# Microfluidic Modeling of Cell−Cell Interactions in Malaria Pathogenesis

**DOI:** 10.1371/journal.ppat.0030099

**Published:** 2007-07-20

**Authors:** Meher Antia, Thurston Herricks, Pradipsinh K Rathod

**Affiliations:** 1 Department of Chemistry, University of Washington, Seattle, Washington, United States of America; 2 Department of Pathobiology, University of Washington, Seattle, Washington, United States of America; Northwestern University Medical School, United States of America

## Abstract

The clinical outcomes of human infections by Plasmodium falciparum remain highly unpredictable. A complete understanding of the complex interactions between host cells and the parasite will require in vitro experimental models that simultaneously capture diverse host–parasite interactions relevant to pathogenesis. Here we show that advanced microfluidic devices concurrently model (a) adhesion of infected red blood cells to host cell ligands, (b) rheological responses to changing dimensions of capillaries with shapes and sizes similar to small blood vessels, and (c) phagocytosis of infected erythrocytes by macrophages. All of this is accomplished under physiologically relevant flow conditions for up to 20 h. Using select examples, we demonstrate how this enabling technology can be applied in novel, integrated ways to dissect interactions between host cell ligands and parasitized erythrocytes in synthetic capillaries. The devices are cheap and portable and require small sample volumes; thus, they have the potential to be widely used in research laboratories and at field sites with access to fresh patient samples.

## Author Summary

With over 500 million clinical cases and 1 million deaths per year, malaria presents a devastating global health problem. Samples from patients with severe disease suggest that binding of malaria-infected red blood cells (iRBCs) to host mammalian cells plays an important role in precipitating blood vessel blockages that can cause organ failure. Yet, some individuals in endemic countries harbor parasites without significant clinical symptoms. To help explore variations in disease outcomes, we developed microfluidic channels that mimic many potential features of severe disease. Synthetic microfluidic channels, with sizes and shapes resembling small capillary networks, were coated with pure host proteins or cultured mammalian cells expressing host ligands. We could therefore simulate binding of iRBCs under high-pressure fluid flow in a realistic capillary environment. By tracking the fate of individual iRBCs, we observed parasite-to-parasite variation in adhesion and an unexpected drop in adhesion when iRBCs passed through the thinnest capillaries. We also showed engulfment of iRBCs by phagocytic cells under fluid flow. The microfluidic devices should serve as powerful field tools for understanding severe malaria because the system is easy to use, requires very small sample volumes, and is portable for on-site analysis of patient samples in the field.

## Introduction

Cellular events that contribute to severe malaria are multi-faceted [1]. Binding of malaria-infected red blood cells (iRBCs) to host endothelium may alter blood flow, affect blood vessel integrity, and contribute to physical blockages of narrow capillaries [2–4]. Loss of red blood cell (RBC) deformability may also contribute to capillary occlusions [5–8]. Activation of endothelial cells can trigger localized inflammatory responses [9–11], and phagocytic activity can lead to anemia through destruction of both infected and uninfected RBCs, particularly in the spleen [12,13]. Since such events can occur in all infected individuals, it is a mystery why some infected patients progress to severe forms of the disease while others harbor high parasitemia and remain symptom free.

Dissection of the molecular basis for variations in malaria pathogenesis relies on many approaches, each with unique advantages but also significant limitations. For instance, human genetics plays an important role in determining disease severity; human RBC disorders, which cause abnormal expression of surface ligands on iRBCs, can protect patients from disease [14]. Animal studies have also contributed significantly to our understanding of severe malaria, but disease pathology in animals can be very different from that in humans [15,16]. Finally, postmortem autopsies provide a direct link to human pathology, but offer limited flexibility for systematic testing of hypotheses for early events leading to pathogenesis [17–20].

In vitro models for malaria pathogenesis can complement studies of human genetics, autopsies, and animal models. In the past, traditional cell binding assays helped identify relevant host cells [21], host proteins [22–25], and parasite proteins [26–28] involved in cytoadherence. Experiments in flow chambers were particularly important in addressing shear stress–dependent adhesion, as seen in capillaries in vivo [29–33]. However, improved in vitro models are needed that more accurately depict iRBC interactions with host cells in small capillaries. Flow chamber dimensions tend to be much larger than typical capillaries where adhered parasitized cells are found; therefore, the peculiarities of blood flow in narrow blood vessels are not accurately mimicked. Flow chambers also offer no design flexibility needed to mimic complex capillary networks found in the microvasculature, where varying shear stresses could be critical in precipitating cytoadhesion or capillary occlusions. Traditional flow chambers are made of gas-impermeable, rigid materials, which do not readily model the elastic, multicellular properties of blood vessels. Finally, the chambers are bulky, cumbersome to transport, and not well suited for work with small biological sample volumes over many hours.

Microfluidic devices made from elastomeric materials can overcome many of the limitations posed by bulk flow chambers. The fabrication techniques have been specifically developed to engineer devices of diverse shapes with micron-sized dimensions. Thus microfluidics allows for flow experiments in channels that have the same dimensions as capillaries in the microvasculature, using very small sample volumes. We recently showed that a 20-μm microfluidic channel with a 2-μm constriction was able to mimic certain aspects of the physical blockage of a narrow capillary by an infected erythrocyte, even in the absence of any host cells [7]. However, the onset of disease in infected individuals is more complicated than capillary obstruction by rigid erythrocytes. Here, we show how to integrate the adhesive interactions between host ligands and parasitized erythrocytes with the unique effects of blood flow in capillary-like environments. We also show that host–parasite interactions over long time periods can be studied in microfluidic devices by demonstrating phagocytic responses to infected erythrocytes under flow conditions. The addition of such complexity to microfluidic systems opens up the potential to use the devices in the field to explore the highly individual responses to malaria infections.

## Results/Discussion

### The Microfluidic System

To mimic blood flow and cytoadherance of infected erythrocytes in capillaries, microfluidic channels of a variety of shapes and sizes were fabricated, including straight, 50-μm-wide channels, channels with narrow, 4-μm constrictions in them, and bifurcating channels that resembled a network of capillaries. The use of polydimethylsiloxane (PDMS) to create the molds that form the channel walls and ceilings is described in the Methods section and is supported by well established chemistry [34]. In physical appearance, the PDMS devices are approximately 3 cm long, 1 or 2 cm wide, and 0.5–1 cm tall. The ends of the channel are perforated with plastic tubing to allow flow to and from the channels. A syringe is connected to the outlet tubing to generate negative pressure across the channel, and a digital manometer is attached through a T-junction to measure this pressure (see Methods for more details). This configuration requires no external pumps, is easy to transport, and can be mounted on practically any inverted microscope for flow measurements. Because the PDMS is irreversibly sealed to the glass substrate, the channels can withstand very high pressures—greater than 8 kPa across the channels—without any leakage. Typical pressure drops across the channels used in subsequent experiments are comparable with what has been measured in vivo for small capillaries. (For example, a 1-kPa pressure drop was measured across a capillary in the cat mesentery [35].) Lower pressures (below 0.2 kPa) are obtained by adjusting the height of the column of fluid in the inlet reservoir of the channel. The elasticity of PDMS (Young's modulus ∼ 750 kPa) resembles that of many blood vessels in vivo (40 or 1,200 kPa, depending on the type of vessel and age) [36,37]. The Methods section describes the approaches used to coat the floor of the channels with either purified protein ligands known to be important in cytoadherance or mammalian cells expressing such ligands.

Fluid flow in microfluidic channels with at least one dimension less than 100 μm is well understood. The flow is laminar, has a low Reynolds number, and has a typical parabolic velocity profile, with the maximum velocity at the center of the channel [38]. The velocity at different spatial positions in microfluidic channels has been measured in previous experiments using submicron-sized fluorescent beads and was found to be in excellent agreement with predicted velocities [38]. Flow at low Reynolds numbers is entirely reversible and is governed only by the pressure drop across the channel and the viscosity of the fluid. The viscosity of blood can be calculated from the hematocrit. Thus, in our system the direct measurement of the pressure and hematocrit allowed us to calculate other parameters related to the fluid flow, such as average fluid velocity or wall shear stress. Although fluid velocity decreases with increasing hematocrit, the wall shear stress remains unchanged since it depends on the product of the viscosity and the fluid velocity.

To establish our methodology, we used the parasite strain ItG-ICAM-1, which is known to bind intracellular adhesion molecule 1 (ICAM-1), an important ligand mediating cytoadhesion in vivo. Both rolling and stationary adhesion of ItG-ICAM-1 to ICAM-1 under flow conditions have been previously measured at low shear stresses (0.05 Pa and 0.1 Pa) [39]. No previous experiments have measured binding of this strain to CD36, another important receptor that binds iRBCs in vivo; however, other ICAM-binding parasite strains are known to bind both ICAM-1 and CD36 [31]. Variations in binding to receptors between different strains have been previously reported, although the qualitative behavior is expected to be similar [39]. Our stocks of ItG-ICAM-1 were regularly selected for binding to purified ICAM-1 prior to introduction in channels [40].

### Adhesion to ICAM-1

Adhesion to ICAM-1 is important for malaria pathogenesis in vivo. ICAM-1 may be particularly important for mediating cytoadhesion in the brain, since immunohistochemical studies have shown that it is upregulated in the cerebral vasculature in fatal malaria cases [41]. Evidence also suggests that without ICAM-1, binding to the endothelium under flow conditions is impaired [30]. Although previous work has shown that ICAM-1 works synergistically with other receptors to mediate stable adhesion to the endothelium [30,33], we provide the first evidence to our knowledge that ICAM-1 alone may be able to mediate stable adhesion in a microfluidic environment.

**Figure 1:**
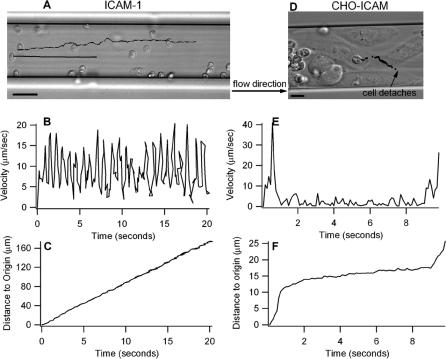
Trajectories of iRBCs Rolling on ICAM-1 (A−C) iRBCs rolling on purified protein. RBC solution at 5% hematocrit and 7% parasitemia was flowed at different pressures through a channel functionalized with ICAM-1, as described in the Methods section. At all measured pressures, 86% of cells that adhered to the surface rolled rather than remained stationary. Of cells that rolled, 99% continued rolling for hundreds of microns rather than arresting on the surface or detaching. (A) Dots mark the spatial position of sample iRBCs every 0.1 s. (B) Instantaneous velocity of iRBCs. (C) Distance to origin over 20 s of rolling. At the high pressures shown here (3 kPa), iRBCs on ICAM-1 rolled in a jerky, stepwise fashion with brief, periodic velocity minima occurring approximately every 0.7 s. See also Video S1. (D−F) iRBCs rolling on mammalian cells expressing ICAM-1 (CHO-ICAM). CHO-ICAM were seeded in channels as described in the Methods section and grown to confluence under continuous flow conditions for 2 d. RBC suspensions at 5% parasitemia and 10% hematocrit were flowed through the channels at various pressures. (D) Dots mark the spatial position of a typical iRBC every 0.1 s. (E) iRBC instantaneous velocity. (F) Distance to origin of a rolling iRBC at an applied pressure of 2 kPa. On CHO-ICAM, iRBCs move sporadically, often coming to a complete halt before starting to roll again, usually deviating significantly from a straight path. Several iRBCs remain statically adhered and do not roll. Scale bars = 10 μm. See also Video S2.

Adhesion of iRBCs to purified ICAM-1 was confirmed in our 50-μm wide × 29-μm tall microfluidic channels even under physiologically relevant shear stresses (applied pressures: 0.5–5 kPa; corresponding shear stress: 0.2–2.5 Pa; [42–44]). These shear stresses are about an order of magnitude higher than shear stresses reported in previous adhesion experiments [31,33,39]. At all measured shear stresses, infected erythrocytes displayed rolling behavior on purified ICAM-1 adsorbed to the channels (Figure 1A−1C and Video S1). Fluorescence labeling confirmed that all rolling or attached RBCs were infected. Large numbers of uninfected erythrocytes flowed past the attached cells, usually without knocking them off the protein-coated surface. At all measured pressures, about 86% of iRBCs that interacted with the surface-adsorbed ICAM-1 rolled rather than remained stationary. Finally, at all pressures, ∼99% of cells that rolled continued rolling for as long as they were followed along the length of the channel (typically 180 μm), rather than arresting on the surface or detaching. Indeed, several cells were observed to roll for several millimeters (over 4 min) without stopping or detaching from the surface, in agreement with previous published results at lower wall shear-stress values [31]. Trajectories of individual rolling iRBCs on ICAM-1 showed that the rolling occurred in a jerky, stepwise manner at all pressures, with periodic changes in velocity. In control experiments, uninfected cells, or erythrocytes infected with non-adherent strains of parasites such as unselected 3D7 or HB3, showed no adhesion to the ICAM-1 surfaces. On purified CD36, only stationary adhesion was observed at pressures below 1 kPa, while both rolling and stationary adhesion were observed at higher pressures [31].

We compared the adhesion of iRBCs under flow conditions to adsorbed ICAM-1 in the presence and absence of soluble ICAM-1. At a pressure of 2 kPa, we found that soluble ICAM-1 inhibited adhesion of ItG-ICAM-1 by up to 85%. Using the microfluidic system, we performed this adhesion inhibition experiment using less than 50 μl of fluid. The use of small volumes of fluid for such experiments will greatly facilitate testing of potential drug or vaccine candidates that block adhesion.

Adhesion was also studied in synthetic microcapillaries seeded with mammalian CHO cells expressing ICAM-1 (CHO-ICAM) and grown to confluence over 2 d. In contrast to behavior on cell-free ICAM-1 ligand, the majority of iRBCs exhibited stationary adhesion on CHO-ICAM (at 0.1 kPa, as well as 3 kPa). Those iRBCs that did roll on CHO-ICAM displayed sporadic behavior, showing large variations in their instantaneous rolling velocities, sometimes coming to a complete halt for several seconds and sometimes detaching from the surface (Figure 1D−1F and Video S2). In control experiments, uninfected cells or erythrocytes infected with unselected 3D7 or HB3 parasites showed no binding to the CHO-ICAM cells. Stationary adhesion was observed on CHO cells expressing CD36 at pressures below 1 kPa, while an increasing fraction of iRBCs rolled at higher pressures, similar to purified CD36 receptor adsorbed to the channels (unpublished data).

The difference in binding to pure ligand versus ligand expressed on mammalian cells was not previously seen in bulk flow chambers that compared rolling of iRBCs on purified ICAM-1 and HUVECs—cells that primarily express ICAM-1 [31]. Since ICAM-1–transfected CHO cells do not express any other known cytoadherence receptors in abundance, it is unlikely that the stable binding we observed on CHO cells is mediated by additional known proteins such as CD36 or thrombospondin [45,46]. Previous computational and experimental work has shown that the presence of cells in a micron-sized channel can greatly alter the flow microenvironment [47]. Therefore, the stable adhesion of iRBCs to CHO-ICAM may be a direct consequence of such flow alterations. The ability of microfluidic devices to support both pure ligands and ligand-expressing human cells in culture, under high shear stress, should facilitate a detailed understanding of the role of the ligand environment in precipitation of capillary blockage in the microvasculature.

### Variations in Rolling Velocities

Even at high pressures in microchannels, iRBCs carrying the ITG strain of Plasmodium falciparum displayed remarkable “meta-stable” attachment to protein surfaces. iRBCs continued to roll on host ligands at pressures as high as 5 kPa without detaching from the surface. This suggests that an interesting and important molecular mechanism promotes such behavior, because in the absence of a compensating mechanism, most cells that roll on substrates are expected to increase their rolling velocities in response to increasing shear stresses and eventually detach from the surface [48]. Furthermore, the ligand ICAM-1, when compared with another important cytoadhesion ligand, CD36, imposed different rolling properties on different iRBCs within a population.

**Figure 2:**
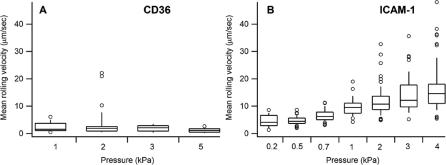
Changes in Rolling Velocities of iRBCs on Purified CD36 and ICAM-1 Box and whisker plots are generated from tracking the average velocities of populations of *individual cells* rolling either on recombinant CD36 or on recombinant ICAM-1. The top and bottom of the box denote the 75th and 25th percentiles of the population, respectively, and the top and bottom of the whiskers denote the 90th and 10th percentiles, respectively. Outliers are marked with open circles. (A) Stabilization of rolling velocities of iRBCs on CD36. At pressures where rolling is observed on CD36, average rolling velocities of most cells remain stable at between approximately 1 and 3 μm/s. Difference between rolling velocities at all pressures was not statistically significant (ANOVA, *p* > 0.01). A few outliers are observed with higher rolling velocities (up to 22 μm/s) at 2 kPa. (B) Variation in rolling velocity on ICAM-1 at different pressures. Populations of iRBCs on ICAM-1 at different pressures showed inhomogeneity of variances (Levene's test, *F* = 12); thus, statistical significance of differences in means could not be evaluated. However, box and whisker plots show only a gradual increase in rolling velocity of most infected cells at higher flow pressures, and only higher velocity rollers increase rolling velocity in direct proportion to increased fluid pressures. At 4 kPa, the highest rolling velocity increased to 45 μm/s, from 32 μm/s at 3 kPa and 26 μm/s at 2 kPa. In contrast, the median rolling velocity only increased to 14.6 μm/s at 4 kPa from 12.1 μm/s at 3 kPa, 10.7 μm/s at 2 kPa, and 9.5 μm/s at 1 kPa.

To illustrate how rolling velocities responded to increasing pressure, we tracked individual iRBCs in a population rolling on either purified CD36 or ICAM-1. On purified CD36, significant rolling required pressures higher than 1 kPa. As pressure increased beyond 1 kPa, iRBCs showed no significant increases in the mean rolling velocities (Figure 2A). A similar analysis of individual iRBCs rolling on ICAM-1 revealed different and even more complex behavior. First, the average rolling velocities on ICAM-1 were higher than those on CD36. In addition, on ICAM-1, populations of iRBCs showed significant differences in the *variances* of rolling velocities at different pressures, indicating that all cells within a population did not roll in an identical fashion. A large proportion of iRBCs appeared to stabilize adhesion and did not show proportional changes in velocity with increasing pressure, particularly at lower pressures. This agrees with previous work that showed rolling velocities for ICAM-1 did not change when shear stress was increased [39]. However, at high pressures, a fraction of iRBCs did indeed increase rolling velocities with increasing pressures (Figure 2B).

The increase in rolling velocities of some cells but not others was not a result of the parabolic fluid velocity profile in a microchannel. First, the measured velocities showed no correlation with the spatial position of iRBCs in the channel; many iRBCs in the same part of the channel had different velocities. Second, all velocity measurements were taken at least 10 μm from the channel walls to exclude any RBCs that may be affected by interactions with the wall. For the aspect ratio used in our devices, the maximum variation in velocity attributable to the parabolic flow profile is approximately 25% [38]. In contrast, the differences in velocity between rolling iRBCs at a particular pressure was typically much greater and increased with increasing pressures. Finally, iRBCs with large variations in rolling velocities were not seen on CD36, indicating that the velocity profile in the channel has a negligible effect on the rolling velocity.

The plateau in rolling velocities of iRBCs at increasing pressure is qualitatively similar to the stability of leukocyte-rolling velocities on selectins at a wide range of shear stresses, both in vivo and in vitro [49,50]. For leukocytes, this has been attributed to a shear-dependent increase in the number of receptor–ligand bonds per rolling step, to compensate for the predicted increase in receptor-ligand dissociation [51]. Cellular characteristics like deformability also contribute to the stabilization of rolling velocities displayed by leukocytes [52]. Similar mechanisms could explain stabilization of rolling velocities for the present iRBC–protein interactions.

Stabilization of rolling velocities of iRBCs on host ligands could have clinical significance. Regulated rolling on capillaries in vivo may allow iRBCs to evenly sample the endothelium, independent of changing dimensions of the blood vessels and the accompanying changes in wall shear stress. Slightly enhanced stabilization of rolling velocities, even in a subpopulation of infected cells, could thus play an important role in promoting accumulation of iRBCs in capillaries.

### Adhesion in Branched Channels

**Figure 3:**
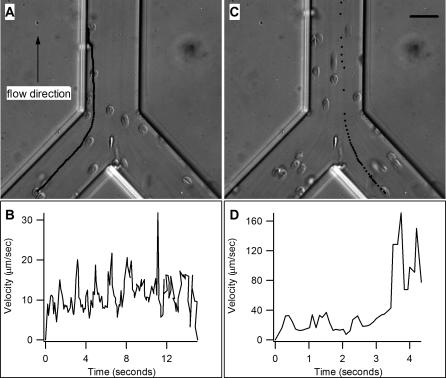
Rolling iRBCs Merging into a Single Channel from a Bifurcation The branching channel was functionalized with ICAM-1. (A, C) Dots represent the spatial position of two differently behaving, rolling iRBCs every 0.1 s at 3 kPa applied pressure. (B) Instantaneous velocity of a rolling cell pictured in (A). The iRBC approaches the fork in the channel after approximately 8 s, but shows no change in rolling velocity. (D) Instantaneous velocity of a rolling iRBC pictured in (C). The iRBC is rolling with a higher velocity than the one pictured in (A) and approaches the fork after approximately 3 s. The iRBC continues rolling in the straight portion of the channel, albeit at a much higher velocity. Scale bar = 25 μm. See also Video S3.

**Figure 4:**
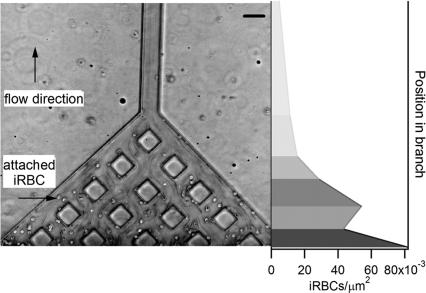
Preferential Attachment of iRBCs in Regions of Lower Fluid Shear Stress In a network of capillaries coated with CD36, a larger number of iRBCs attach in the branches, where shear stress is lower than in the main channel. Applied pressure is 1 kPa across the entire network, making the pressure in individual branches low enough for iRBCs to bind to CD36 in a stationary manner rather than rolling. Image was taken after approximately 10 min of continuous flow. Scale bar = 50 μm.

Branching capillaries are natural sites in the circulatory system where changes in blood flow patterns can lead to alterations in wall shear stress [43]. Microfluidic technology enabled us to fabricate a device that mimicked branching capillaries, with a main channel connected to a network of secondary channels (Figures 3 and 4). Given the complex responses of rolling iRBCs to changes in flow pressures, we hypothesized that individual iRBCs in such branched channels functionalized with ICAM-1 would show different rolling behavior at the sites of shear-stress changes. iRBCs displayed continued rolling behavior upon encountering a fork and followed the path dictated by the bulk fluid flow (Video S3). Velocities of rolling erythrocytes upon reaching the branches, however, varied from cell to cell and displayed one of two patterns. Some cells did not change rolling velocities as they moved from the bifurcating branch into the main artery of the channel, despite the increase in wall shear stress (Figure 3B). Yet, other cells displayed significant increases in rolling velocity (Figure 3D). Flow in microfluidic channels is entirely reversible and depends only on the pressure difference between the entrance and exit of the channel [38]. Thus, iRBCs flowing in the reverse direction—from the main artery into the branch of the channel—displayed the same behavior.

Branched channels were also used to determine whether the accumulation of stably adhering iRBCs was dependent on the shear stress in a simulated capillary network. In a channel functionalized with CD36, at pressures where primarily static adhesion is observed, we found increased accumulation of iRBCs in the branches of a model capillary network relative to the main artery (Figure 4).

These studies demonstrate that microfluidic devices can be fabricated to identify and possibly select cell types that will most likely stabilize rolling upon encountering lower shear stresses. They also show how changing shear stresses due to the shape of a capillary in vivo may be critical in determining where cytoadhesion will likely occur. Clearly, sequestration of infected erythrocytes may depend on the *location* of host cells with adhesive ligands in the microvasculature, as well as the type and quantity of expressed ligands and the nature of the individual iRBCs. Future microfluidic studies can be designed to explore the influence of ligand concentrations, or even mixtures of ligands on cytoadherance by RBCs harboring different parasite clones.

### Adhesion in Constricted Channels

**Figure 5:**
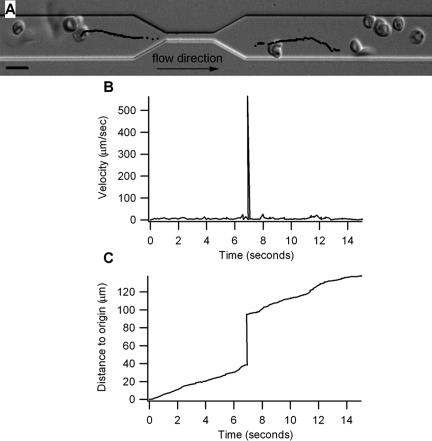
Passage of iRBCs through a Constricted Channel Functionalized with ICAM-1 (A) Tracking the movement of an iRBC in a narrowing constriction. Dots mark the spatial position of a typical iRBC every 0.1 s before and after passage through the constriction. (B) Instantaneous velocity of iRBC. Before reaching the constriction, the iRBC moved with the typical jerky, stepwise motion of rolling iRBCs. The velocity spiked each time an iRBC passed through the constriction. (C) Distance from origin of an iRBC over time. The iRBC moved uniform distances over each time step before reaching the constriction. The erythrocyte then moved through the entire distance of the constriction within a single time frame of 0.1 s. Scale bar = 10 μm. See also Video S4.

Erythrocytes in the microvasculature can encounter capillaries with dimensions smaller than the RBC diameter. Historically, such constrictions have been thought to interfere with circulation of rigidified iRBCs [3,6,10]. Intuitively, it would appear that coating of constricting capillaries with adhesive proteins would further promote blockage. To test this simple hypothesis, we designed an ICAM-1–coated channel that was 5-μm tall and began with a 20-μm width that constricted to 5 μm before returning to 20 μm (Figure 5A). The tight constriction was just wide enough to permit a normal RBC, as well as infected erythrocytes, to squeeze through in the absence of ligand [7].

The behavior of rolling iRBCs as they approached and passed through 5-μm-wide ligand-coated constrictions dramatically illustrated how microfluidic technology permits experiments that would be impossible in conventional flow chambers. As the rolling iRBCs entered the constriction, they briefly ceased rolling and actually accelerated through the pore. This was recorded as a jump in the distance traveled over the length of the constriction and a corresponding spike in the iRBC velocity (Figure 5B and Video S4). Upon exiting the 5-μm constriction, the iRBCs efficiently reattached on the other end and continued rolling at a velocity similar to that before entering the constriction.

The rapid traverse of iRBCs in the narrow part of the channel was not due to uneven coating of ICAM-1 on the channel walls; a fluorescently labeled antibody to ICAM confirmed the presence of the ICAM-1 protein throughout the channel, including in the 5-μm constriction. The decreased interaction of iRBCs with adhesive proteins in confined spaces could be due to one of two other reasons. The large pressure drop across the narrow constriction could create wall shear stresses that readily override the adhesion capabilities of iRBCs. Alternatively, the inability of iRBCs to roll in the confined environment could reduce their affinity for adsorbed ligands. Regardless, the presence of the adhesive protein on the surface of the narrow channels did not augment the formation of obstructions within RBC-sized channels. These results suggest that, unless additional events are in play, the narrowest capillaries in vivo may not necessarily be the first to become obstructed with iRBCs.

### Adhesion to Macrophages under Flow

**Figure 6:**
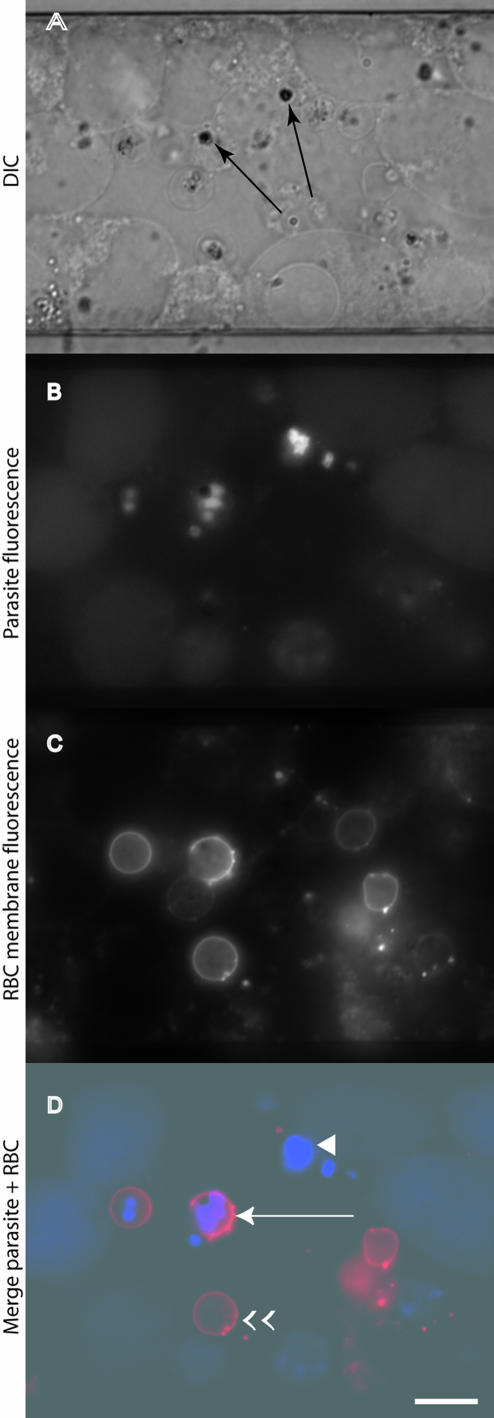
Phagocytosis of iRBCs under Flow (A) Differential interference contrast image of RAW macrophages in a 50-μm channel after lysis of attached RBCs. Arrows show the malarial pigment, hemozoin. (B) Fluorescence image of parasite nuclei ingested by macrophages. (C) DiIC staining of RBC membranes. (D) Parasite DNA fluorescence (blue) and RBC membranes (red) merged. Long arrow shows ingestion of entire iRBC, arrowhead shows ingestion of only the parasite, and double arrowhead shows ingestion of RBC without parasite. Scale bar = 10 μm.

Clearance of parasites from a naive infected individual is largely dependent on the phagocytosis of iRBCs by macrophages in the spleen. To build on experiments on phagocytosis of iRBCs by macrophages in static cultures [53,54], we studied the interactions between iRBCs and macrophages under shear flow in a 50-μm-wide channel for over 20 h. iRBCs rolled slowly on RAW macrophages and finally halted, similar to the behavior exhibited on CHO-ICAM cells. Fluorescently labeled parasite DNA confirmed that over 90% of bound RBCs were parasitized. After 30 min of RBC flow, the attached cells were subjected to continuous media flow, without additional RBCs. After 2 h, 65% of iRBCs remained attached to the macrophages. Of these, approximately half were internalized after 20 h under continuous media flow (Figure 6).

Phagocytosis of infected erythrocytes under shear flow occurred in one of several ways (Figure 6). Some macrophages ingested parasites together with the intact RBC membrane, as judged by simultaneous fluorescence labeling of the RBC membrane and parasite DNA. However, in many cases, the parasites were internalized without any accompanying RBC membrane, reminiscent of in vivo observations of parasite “pitting” by macrophages [55–57]. We also saw evidence of phagocytosis of uninfected cells, which could represent the phagocytosis of a previously “pitted” erythrocyte, an aging uninfected RBC, an uninfected RBC tagged with parasite proteins [58], or a residual erythrocyte ghost from a ruptured schizont. This observation agrees with autopsy data that show phagocytosis of non-parasitized erythrocytes in large numbers in the spleen [59]. Finally, our microfluidic system revealed hemozoin internalization in the macrophages, not only in the same cell as a fluorescent parasite but also in some cells that contained no parasites. Again, this is consistent with human autopsy data [59].

### Conclusions

Microfluidic devices offer a powerful new opportunity to study malaria pathogenesis and other human diseases that involve the microvasculature. The present laboratory-based applications of this advancing technology illustrate the types of questions in malaria pathogenesis that may be addressed with microfluidics. Since the devices are portable and require mere microliter volumes of samples, future applications should be possible at field sites, using matched patient samples. Such studies could include parasitized blood, serum, platelets, antibodies, phagocytic cells, and possibly biopsied host samples. We expect that the most valuable insights into the causes of severe malaria will arise from detailed studies of variations in human and parasite samples at field sites.

Technically, even though the fabrication of the silicon master for a specific experimental application requires an experienced materials science engineer and a specialized clean room facility, subsequent production of dozens of PDMS devices from a common master is inexpensive and easy to learn. As illustrated, the soft lithography methodology allows for the design of channels of a wide variety of shapes and very small sizes, and the gas-permeable PDMS polymer readily accommodates long-term cell growth of multiple cell types in channels. The microfluidic devices can be mounted on a microscope, and data on single cells can be collected as still photos or as movies on a personal computer for further detailed analysis. In addition to their use in field sites, we expect the devices to be popular in standard research laboratories where access to traditional flow adhesion apparatus is either unavailable or impractical due to the large volumes of sample needed.

## Materials and Methods

### Microfluidic channel fabrication.

Microfluidic silicon masters were fabricated using standard photolithographic techniques [7,34]. Briefly, channel patterns were created on a quartz-chrome mask (Photo Sciences, http://www.photo-sciences.com/) and imprinted on silicon wafers (Montco Silicon Technologies, http://www.silicon-wafers.com/) using photoresist. Relief features were etched using the Bosch deep reactive ion etch process (Oxford Instruments, http://www.oxford-instruments.com/). To make the elastomeric microfluidic devices from the silicon masters, PDMS (Dow Corning, http://www.dowcorning.com/) was poured over the silicon master, cured, and then cut from the master and irreversibly sealed to a glass coverslip after oxygen plasma treatment (Harrick Scientific Products, http://www.harricksci.com/). Access to the channels was possible through a 5-mm hole punched on one end of the channel to form a reservoir for the sample, with a smaller hole punched by a 21-gauge needle at the other end, into which polyethylene tubing (PE20) attached to a syringe could be inserted. Pressure was controlled by manually adjusting the plunger on a syringe and was measured with a digital manometer inserted between the tubing and the syringe. Flow rates were calculated from applied pressures using Poiseuille's equation for flow in a channel with a rectangular cross section [60], from which wall shear stresses could be calculated [31].

### Malaria parasites.

The ICAM-1–adherent laboratory line of P. falciparum (ItG-ICAM-1) was used for all assays and was a kind gift from Joseph Smith (Seattle Biomedical Research Institute, http://www.sbri.org/). Parasites were periodically selected by passage over recombinant ICAM-1 protein (R&D Systems, http://www.rndsystems.com/) spotted on Falcon 1007 petri dishes. Parasites were cultured in human RBCs according to standard protocols. Unsynchronized cultures were used for all experiments, with parasitemia (assessed by Giemsa-stained blood smears) ranging between 5% and 17%. For all adhesion assays, parasite cultures were washed twice in pre-warmed binding medium, consisting of RPMI 1640 with 0.5% BSA (pH 7.2) and resuspended in the binding medium at a hematocrit of 2%–10%.

### Mammalian cell cultures.

CHO cells transfected with CD36 were a gift from Joseph Smith, and CHO cells transfected with ICAM-1 were obtained from ATCC (http://www.atcc.org/). Both cells lines were cultured in F-12K nutrient mixture supplemented with 10% fetal bovine serum, 5% penicillin-streptomycin, and 0.25 mg/ml Geneticin (Invitrogen, http://www.invitrogen.com/). RAW macrophages were cultured in DMEM supplemented with 10% fetal bovine serum and 5% penicillin-streptomycin (Invitrogen).

### Functionalization of microfluidic channels with pure ligands.

The channels were first rinsed continuously with a flow of ethanol for about 10 min, followed by rinsing with a 4% solution of aminopropylethoxysilane (APES; Sigma-Aldrich, http://www.sigmaaldrich.com/) in ethanol for about 15 min, to prepare the glass surface for protein adsorption. Solutions of either ICAM-1 or CD36 in MilliQ water (both at concentrations of 50 μg/ml, R&D Systems) were introduced into the channels at low flow rates for approximately 2 h at 37 °C. Channels were then blocked for 2 h with a 2% BSA solution. A similar protocol was previously used to functionalize glass microslides with both CD36 and ICAM-1 [61]. To ensure reproducibility of the protein surface, we used concentrations of CD36 and ICAM-1 that are well above those that saturate the surface.

For adhesion blocking with soluble ICAM-1, 3 μl of packed RBCs enriched to 30% parasitemia using Plasmion plasmagel were incubated in 50 μl of ICAM-1 at a concentration of 50 μg/ml for 15 min at 37 °C. The RBC solution was then flowed through the microfluidic chamber at a pressure of 2 kPa for 12 min, after which the number of attached cells were counted over at least eight different fields of view. The number of attached cells was compared with the number obtained by flowing into the channel an equivalent concentration of iRBCs that were not exposed to soluble ICAM-1 at the same pressure for the same time.

### Mammalian cells in microfluidic channels.

Channels were first incubated with the appropriate cell culture media for approximately 1 h at 37 °C prior to introducing cells. About 200 μl of cells in media were pipetted into the channel reservoir at a concentration of about 5 million cells/ml. The cells were pulled into the channel and allowed to settle. Unattached cells were rinsed away and the process was repeated to achieve an attached cell density that would support the growth of a confluent monolayer. Cells in the channels were grown under continuous fluid flow for up to 3 d and shown to be alive using a fluorescent Live/Dead Cell Vitality Assay (Molecular Probes, http://probes.invitrogen.com/).

### Microscopy.

All imaging of cells and channels was carried out on an inverted fluorescence microscope (Nikon TE200 or TE2000; http://www.nikon.com/), with either a 40× (Plan Fluor, 0.75 NA, Nikon) or an oil immersion 100× (Plan Fluor, 1.3 NA, Nikon) objective. Movies and images of infected erythrocytes in channels were captured on a high-sensitivity CCD camera (a Hamamatsu Digital Camera C4742–98, Hamamatsu CCD Camera [video] C2400, or a Photometrics CoolSnap ES [Roper Scientific, http://www.roperscientific.com/]). Image and movie acquisition was with Metamorph Imaging System (Molecular Devices, http://www.moleculardevices.com/). A home-built temperature-controlled stage maintained a 37 °C environment for the experiments with live mammalian cells in the channels.

### Measurement of rolling velocities of infected erythrocytes.

Movies of rolling iRBCs were analyzed using the tracking software on the Metamorph Imaging System. *x* and *y* coordinates of cells at each acquisition frame (every 0.1 s) were recorded, from which instantaneous velocities, average velocities, and distances from the origin were calculated. Data were further analyzed using Igor Pro software (Wavemetrics, http://www.wavemetrics.com/). Statistical analysis was performed with the Igor Pro ANOVA package.

### Phagocytosis assay.

RAW macrophages were seeded and grown in 50 μm × 29 μm channels and iRBC cultures introduced at a pressure of 0.1 kPa. Channels were kept overnight in an incubator at 37 °C and 5% CO_2_, with the flow rate maintained by gravity. Infected erythrocytes were counted by taking an average of approximately 20 random fields of view of the attached macrophages in the channel. Phagocytosis was measured after lysis of attached erythrocytes with cold water, as previously described [53,54].

## Supporting Information

Video S1 Adhesive Rolling of iRBCs on an ICAM-Coated Channel Surface (2.4 MB MPG)

Video S2 Adhesion of iRBCs to ICAM-CHO Cells Cultivated on Channel Floor (2.9 MB MPG)

Video S3 Rolling iRBCs at Bifurcation Points with Changing Shear Forces (622 KB MPG)

Video S4 Loss of Adhesion of iRBCs on Functionalized Constrictions in Microfluidic Channels (491 KB MPG)

## Abbreviations

CHO-ICAM: mammalian CHO cells expressing ICAM-1
ICAM-1: intracellular adhesion molecule 1
iRBC: infected red blood cell
PDMS: polydimethylsiloxane
RBC: red blood cell

## References

[ppat-0030099-b001] Miller LH, Baruch DI, Marsh K, Doumbo OK (2002). The pathogenic basis of malaria. Nature.

[ppat-0030099-b002] Brown H, Hien TT, Day N, Mai NT, Chuong LV (1999). Evidence of blood–brain barrier dysfunction in human cerebral malaria. Neuropathol Appl Neurobiol.

[ppat-0030099-b003] Dondorp AM, Pongponratn E, White NJ (2004). Reduced microcirculatory flow in severe falciparum malaria: Pathophysiology and electron-microscopic pathology. Acta Trop.

[ppat-0030099-b004] Turner GD, Ly VC, Nguyen TH, Tran TH, Nguyen HP (1998). Systemic endothelial activation occurs in both mild and severe malaria. Correlating dermal microvascular endothelial cell phenotype and soluble cell adhesion molecules with disease severity. Am J Pathol.

[ppat-0030099-b005] Cranston HA, Boylan CW, Carroll GL, Sutera SP, Williamson JR (1984). Plasmodium falciparum maturation abolishes physiologic red cell deformability. Science.

[ppat-0030099-b006] Nash GB, O'Brien E, Gordon-Smith EC, Dormandy JA (1989). Abnormalities in the mechanical properties of red blood cells caused by Plasmodium falciparum. Blood.

[ppat-0030099-b007] Shelby JP, White J, Ganesan K, Rathod PK, Chiu DT (2003). A microfluidic model for single-cell capillary obstruction by *Plasmodium falciparum–*infected erythrocytes. Proc Natl Acad Sci U S A.

[ppat-0030099-b008] Dondorp AM, Angus BJ, Hardeman MR, Chotivanich KT, Silamut K (1997). Prognostic significance of reduced red blood cell deformability in severe falciparum malaria. Am J Trop Med Hyg.

[ppat-0030099-b009] Grau GE, Taylor TE, Molyneux ME, Wirima JJ, Vassalli P (1989). Tumor necrosis factor and disease severity in children with falciparum malaria. N Engl J Med.

[ppat-0030099-b010] Patnaik JK, Das BS, Mishra SK, Mohanty S, Satpathy SK (1994). Vascular clogging, mononuclear cell margination, and enhanced vascular permeability in the pathogenesis of human cerebral malaria. Am J Trop Med Hyg.

[ppat-0030099-b011] Urquhart AD (1994). Putative pathophysiological interactions of cytokines and phagocytic cells in severe human falciparum malaria. Clin Infect Dis.

[ppat-0030099-b012] Engwerda CR, Beattie L, Amante FH (2005). The importance of the spleen in malaria. Trends Parasitol.

[ppat-0030099-b013] Ferrant A (1983). The role of the spleen in haemolysis. Clin Haematol.

[ppat-0030099-b014] Fairhurst RM, Wellems TE (2006). Modulation of malaria virulence by determinants of Plasmodium falciparum erythrocyte membrane protein-1 display. Curr Opin Hematol.

[ppat-0030099-b015] Kawai S, Aikawa M, Kano S, Suzuki M (1993). A primate model for severe human malaria with cerebral involvement: Plasmodium coatneyi–infected Macaca fuscata. Am J Trop Med Hyg.

[ppat-0030099-b016] Pettersson F, Vogt AM, Jonsson C, Mok BW, Shamaei-Tousi A (2005). Whole-body imaging of sequestration of *Plasmodium falciparum* in the rat. Infect Immun.

[ppat-0030099-b017] Pongponratn E, Riganti M, Punpoowong B, Aikawa M (1991). Microvascular sequestration of parasitized erythrocytes in human falciparum malaria: A pathological study. Am J Trop Med Hyg.

[ppat-0030099-b018] MacPherson GG, Warrell MJ, White NJ, Looareesuwan S, Warrell DA (1985). Human cerebral malaria. A quantitative ultrastructural analysis of parasitized erythrocyte sequestration. Am J Pathol.

[ppat-0030099-b019] Silamut K, Phu NH, Whitty C, Turner GD, Louwrier K (1999). A quantitative analysis of the microvascular sequestration of malaria parasites in the human brain. Am J Pathol.

[ppat-0030099-b020] Taylor TE, Fu WJ, Carr RA, Whitten RO, Mueller JS (2004). Differentiating the pathologies of cerebral malaria by postmortem parasite counts. Nat Med.

[ppat-0030099-b021] Udeinya IJ, Schmidt JA, Aikawa M, Miller LH, Green I (1981). Falciparum malaria-infected erythrocytes specifically bind to cultured human endothelial cells. Science.

[ppat-0030099-b022] Berendt AR, Simmons DL, Tansey J, Newbold CI, Marsh K (1989). Intercellular adhesion molecule-1 is an endothelial cell adhesion receptor for Plasmodium falciparum. Nature.

[ppat-0030099-b023] Fried M, Duffy PE (1996). Adherence of Plasmodium falciparum to chondroitin sulfate A in the human placenta. Science.

[ppat-0030099-b024] Oquendo P, Hundt E, Lawler J, Seed B (1989). CD36 directly mediates cytoadherence of Plasmodium falciparum parasitized erythrocytes. Cell.

[ppat-0030099-b025] Roberts DD, Sherwood JA, Spitalnik SL, Panton LJ, Howard RJ (1985). Thrombospondin binds falciparum malaria parasitized erythrocytes and may mediate cytoadherence. Nature.

[ppat-0030099-b026] Waterkeyn JG, Wickham ME, Davern KM, Cooke BM, Coppel RL (2000). Targeted mutagenesis of Plasmodium falciparum erythrocyte membrane protein 3 (PfEMP3) disrupts cytoadherence of malaria-infected red blood cells. EMBO J.

[ppat-0030099-b027] Crabb BS, Cooke BM, Reeder JC, Waller RF, Caruana SR (1997). Targeted gene disruption shows that knobs enable malaria-infected red cells to cytoadhere under physiological shear stress. Cell.

[ppat-0030099-b028] Baruch DI, Gormely JA, Ma C, Howard RJ, Pasloske BL (1996). Plasmodium falciparum erythrocyte membrane protein 1 is a parasitized erythrocyte receptor for adherence to CD36, thrombospondin, and intercellular adhesion molecule 1. Proc Natl Acad Sci U S A.

[ppat-0030099-b029] Nash GB, Cooke BM, Marsh K, Berendt A, Newbold C (1992). Rheological analysis of the adhesive interactions of red blood cells parasitized by Plasmodium falciparum. Blood.

[ppat-0030099-b030] Gray C, McCormick C, Turner G, Craig A (2003). ICAM-1 can play a major role in mediating *P. falciparum* adhesion to endothelium under flow. Mol Biochem Parasitol.

[ppat-0030099-b031] Cooke BM, Berendt AR, Craig AG, MacGregor J, Newbold CI (1994). Rolling and stationary cytoadhesion of red blood cells parasitized by *Plasmodium falciparum:* Separate roles for ICAM-1, CD36 and thrombospondin. Br J Haematol.

[ppat-0030099-b032] Avril M, Traore B, Costa FT, Lepolard C, Gysin J (2004). Placenta cryosections for study of the adhesion of *Plasmodium falciparum–*infected erythrocytes to chondroitin sulfate A in flow conditions. Microbes Infect.

[ppat-0030099-b033] Yipp BG, Anand S, Schollaardt T, Patel KD, Looareesuwan S (2000). Synergism of multiple adhesion molecules in mediating cytoadherence of *Plasmodium falciparum–*infected erythrocytes to microvascular endothelial cells under flow. Blood.

[ppat-0030099-b034] McDonald JC, Duffy DC, Anderson JR, Chiu DT, Wu H (2000). Fabrication of microfluidic systems in poly(dimethylsiloxane). Electrophoresis.

[ppat-0030099-b035] Lipowsky HH, Zweifach BW (1977). Methods for the simultaneous measurement of pressure differentials and flow in single unbranched vessels of the microcirculation for rheological studies. Microvasc Res.

[ppat-0030099-b036] Caro CG, Pedley TJ, Seed WA, Guyton AC (1974). Mechanics of the circulation. Cardiovascular physiology.

[ppat-0030099-b037] Lötters JC, Olthuis W, Veltink PH, Bergveld P (1997). The mechanical properties of the rubber elastic polymer polydimethylsiloxane for sensor applications. J Micromech Microeng.

[ppat-0030099-b038] Brody JP, Yager P, Goldstein RE, Austin RH (1996). Biotechnology at low Reynolds numbers. Biophys J.

[ppat-0030099-b039] Adams S, Turner GD, Nash GB, Micklem K, Newbold CI (2000). Differential binding of clonal variants of Plasmodium falciparum to allelic forms of intracellular adhesion molecule 1 determined by flow adhesion assay. Infect Immun.

[ppat-0030099-b040] Ockenhouse CF, Ho M, Tandon NN, Van Seventer GA, Shaw S (1991). Molecular basis of sequestration in severe and uncomplicated Plasmodium falciparum malaria: Differential adhesion of infected erythrocytes to CD36 and ICAM-1. J Infect Dis.

[ppat-0030099-b041] Turner GD, Morrison H, Jones M, Davis TM, Looareesuwan S (1994). An immunohistochemical study of the pathology of fatal malaria. Evidence for widespread endothelial activation and a potential role for intercellular adhesion molecule-1 in cerebral sequestration. Am J Pathol.

[ppat-0030099-b042] Ngai AC, Winn HR (1996). Estimation of shear and flow rates in pial arterioles during somatosensory stimulation. Am J Physiol.

[ppat-0030099-b043] Malek AM, Alper SL, Izumo S (1999). Hemodynamic shear stress and its role in atherosclerosis. JAMA.

[ppat-0030099-b044] Lipowsky HH, Kovalcheck S, Zweifach BW (1978). The distribution of blood rheological parameters in the microvasculature of cat mesentery. Circ Res.

[ppat-0030099-b045] Ringwald P, Dubois B, Le Bras J, Deloron P (1993). *Plasmodium falciparum:* In vitro models of cytoadherence of infected erythrocytes and an analysis with eight different isolates on different target cells. Exp Parasitol.

[ppat-0030099-b046] Hasler T, Albrecht GR, Van Schravendijk MR, Aguiar JC, Morehead KE (1993). An improved microassay for Plasmodium falciparum cytoadherence using stable transformants of Chinese hamster ovary cells expressing CD36 or intercellular adhesion molecule-1. Am J Trop Med Hyg.

[ppat-0030099-b047] Sugihara-Seki M (2000). Flow around cells adhered to a microvessel wall. I. Fluid stresses and forces acting on the cells. Biorheology.

[ppat-0030099-b048] Chen S, Alon R, Fuhlbrigge RC, Springer TA (1997). Rolling and transient tethering of leukocytes on antibodies reveal specializations of selectins. Proc Natl Acad Sci U S A.

[ppat-0030099-b049] Firrell JC, Lipowsky HH (1989). Leukocyte margination and deformation in mesenteric venules of rat. Am J Physiol.

[ppat-0030099-b050] Atherton A, Born GV (1973). Relationship between the velocity of rolling granulocytes and that of the blood flow in venules. J Physiol.

[ppat-0030099-b051] Chen S, Springer TA (1999). An automatic braking system that stabilizes leukocyte rolling by an increase in selectin bond number with shear. J Cell Biol.

[ppat-0030099-b052] Yago T, Leppanen A, Qiu H, Marcus WD, Nollert MU (2002). Distinct molecular and cellular contributions to stabilizing selectin-mediated rolling under flow. J Cell Biol.

[ppat-0030099-b053] McGilvray ID, Serghides L, Kapus A, Rotstein OD, Kain KC (2000). Nonopsonic monocyte/macrophage phagocytosis of *Plasmodium falciparum–*parasitized erythrocytes: A role for CD36 in malarial clearance. Blood.

[ppat-0030099-b054] Patel SN, Serghides L, Smith TG, Febbraio M, Silverstein RL (2004). CD36 mediates the phagocytosis of Plasmodium falciparum–infected erythrocytes by rodent macrophages. J Infect Dis.

[ppat-0030099-b055] Schnitzer B, Sodeman T, Mead ML, Contacos PG (1972). Pitting function of the spleen in malaria: Ultrastructural observations. Science.

[ppat-0030099-b056] Ash C (2003). Pitting erythrocytes. Science.

[ppat-0030099-b057] Chotivanich K, Udomsangpetch R, McGready R, Proux S, Newton P (2002). Central role of the spleen in malaria parasite clearance. J Infect Dis.

[ppat-0030099-b058] Layez C, Nogueira P, Combes V, Costa FT, Juhan-Vague I (2005). Plasmodium falciparum rhoptry protein RSP2 triggers destruction of the erythroid lineage. Blood.

[ppat-0030099-b059] Pongponratn E, Riganti M, Harinasuta T, Bunnag D (1989). Electron microscopic study of phagocytosis in human spleen in falciparum malaria. Southeast Asian J Trop Med Public Health.

[ppat-0030099-b060] Bao JB, Harrison DJ (2006). Measurement of flow in microfluidic networks with micrometer-sized flow restrictors. AIChE Journal.

[ppat-0030099-b061] Cooke BM, Coppel RL, Nash GB (2002). Preparation of adhesive targets for flow-based cytoadhesion assays. Methods Mol Med.

